# Decoding Visual Pathway Dysfunction with SERF-MEG: A Study in Patients with Optic Neuropathy

**DOI:** 10.3390/bioengineering13060694

**Published:** 2026-06-17

**Authors:** Helei Wang, Yuankun Qi, Yu Lou, Xu Zhang, Xinda Song

**Affiliations:** 1 School of Instrumentation and Optoelectronic Engineering, Beihang University, Beijing 100191, China; wanghelei326@buaa.edu.cn (H.W.); yuankunqi@buaa.edu.cn (Y.Q.); louyu@buaa.edu.cn (Y.L.); 2 Hangzhou National Institute of Extremely-Weak Magnetic Field Infrastructure, Hangzhou 310051, China; 3 State Key Laboratory of Traditional Chinese Medicine Syndrome, Hangzhou 310051, China

**Keywords:** SERF-MEG, optic nerve injury, visual evoked responses, functional connectivity, graph theory, visual cortex

## Abstract

This study aimed to characterize cortical dysfunction and frequency-specific network reorganization following optic nerve injury using spin-exchange relaxation-free magnetoencephalography (SERF-MEG), and to assess the potential of MEG-derived multiscale features as sensitive functional biomarkers for clinical evaluation. In this prospective case–control study, SERF-MEG recordings were acquired during a pattern-reversal visual stimulation paradigm. Time-domain evoked components (M100/M135), global electrophysiological indices, energy-based metrics, and alpha- and beta-band phase-based functional connectivity were extracted. Network topology was quantified using graph-theoretical measures, including global and local efficiency, clustering coefficient, and assortativity. Group-level differences between patients and healthy controls were statistically analyzed. Patients showed significantly reduced M100/M135 amplitudes, prolonged M100 latency, and a lower early-component energy ratio. Functional connectivity was significantly decreased in the alpha and beta bands, accompanied by reduced global and local efficiency, mean strength, and clustering coefficient. Seed-based analyses revealed reduced connectivity predominantly in occipito-parietal and occipito-temporal pathways. SERF-MEG provides sensitive identification of cortical- and network-level functional impairments following optic nerve damage. MEG has significant clinical potential for disease diagnosis and therapy monitoring, providing a novel objective assessment tool for neuro-ophthalmological disorders.

## 1. Introduction

Optic neuritis (ON), ischemic optic neuropathy (ION), and traumatic optic neuropathy (TON) are among the most prevalent disorders in neuro-ophthalmology and may lead to acute or chronic visual impairment, substantially compromising patients [1,2,3]. In systemic neurological diseases such as multiple sclerosis (MS), neuromyelitis optica spectrum disorders (NMOSD), and traumatic brain injury, optic nerve damage is not confined to the anterior visual pathway but may also be accompanied by secondary alterations in the lateral geniculate nucleus (LGN) and visual cortex [4,5]. Understanding how optic nerve injury reshapes cortical function and large-scale brain networks is therefore crucial for accurate functional assessment, prognosis, and therapeutic monitoring.

Currently, visual evoked potentials (VEP) and optical coherence tomography (OCT) are the most frequently utilized clinical procedures for evaluating optic nerve injury. Prolonged P100 latency and reduced amplitude in VEP recordings are well-established electrophysiological markers of visual pathway dysfunction [6], while OCT provides quantitative measurements of retinal nerve fiber layer (RNFL) thickness and is commonly used to assess structural optic nerve damage [7]. However, both techniques have inherent constraints. VEP has limited spatial resolution, is sensitive to external noise, and displays considerable inter-individual variability, which constrains its therapeutic applicability [8]. OCT, on the other hand, largely reflects structural alterations at the optic disc and cannot capture functional variations in the visual pathway [7,9]. They generally reflect peripheral conduction impairments and anatomical abnormalities, but lack the ability to properly describe functional disruptions at the cortical level, variations in neuronal synchronization, and large-scale network reconfiguration.

Magnetoencephalography (MEG), a noninvasive neuroimaging method with both high temporal and spatial resolution, offers a novel strategy for investigating the visual pathway and associated cortical networks [10,11]. MEG provides direct recording of magnetic fields generated by synchronous brain activity with millisecond-level temporal precision [12]. When linked with source modeling and functional connectivity research, MEG enables for systematic characterization of the spatial organization, frequency-specific dynamics, and topological properties of large-scale brain networks [13,14,15]. In contrast to conventional VEP, which primarily relies on local time-domain measures such as amplitude and latency [6], MEG can record early visually evoked responses while simultaneously revealing insights into network-level integration and rearrangement within the visual system. Accumulating evidence suggests that optic nerve injury induces systematic alterations in the topology of visual cortical networks, including changes in network efficiency, clustering, and modular organization, reflecting impaired information integration and potential compensatory reorganization [5,16]. Moreover, the frequency-resolved property of MEG enables differentiation of the functional roles of discrete brain oscillations. Low-frequency alpha and beta rhythms are predominantly involved in feedback processing and top-down regulation within the visual system, whereas higher-frequency gamma oscillations are more closely connected with feedforward information transmission [17,18]. These frequency-specific features are difficult to obtain with standard VEP recordings. With the continual development of functional connectivity measurements and graph-theoretical approaches, MEG has emerged as a valuable tool for explaining cortical and network-level reconfiguration following optic nerve injury [19,20,21,22,23,24].

In recent years, improvements in spin-exchange relaxation-free (SERF) atomic magnetometers have propelled the development of a new generation of MEG devices. Compared with classic superconducting quantum interference device (SQUID)-based MEG, SERF-MEG does not require cryogenic cooling with liquid helium, boasts a more compact system architecture, and achieves similar or even better sensitivity within critical frequency ranges [25,26,27,28]. These technical advantages considerably cut installation and operational costs, enabling flexible deployment in clinical scenarios, and improve the viability of MEG for clinical translation. Consequently, SERF-MEG is a promising tool for functional assessment of optic nerve illnesses and other neuro-ophthalmological diseases.

Despite these improvements, most present clinical research continue to depend on VEP or functional magnetic resonance imaging (fMRI), and data relating optic nerve injury to cortical responses and network reconfiguration utilizing MEG remains restricted [5,6,16,29]. Therefore, the primary aim of the present experiment was to evaluate the clinical utility of MEG in detecting and quantifying optic nerve dysfunction and in characterizing its impact on cortical responses and network structure. Using healthy controls as a reference, we carefully evaluated MEG features across patients with ON, ION, and TON. The principal subjects of inquiry included graph-theoretical measurements, frequency-specific functional connectivity, time-domain responses (M100/M135), and global field power (GFP). In order to determine whether MEG can be used as a sensitive and clinically useful tool for evaluating optic nerve function, elucidating cortical changes associated with optic nerve damage, and providing objective evidence to support clinical diagnosis and translational applications, we conducted this extensive evaluation.

## 2. Materials and Methods

### 2.1. Research Design and Ethical Authorization

**Figure 1:**
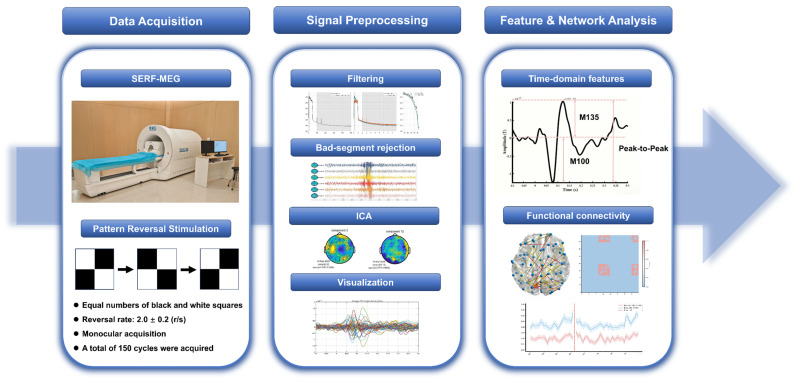
The experimental workflow for SERF-MEG-based visual pathway assessment. There are three primary steps in the pipeline: (i) Data acquisition, which includes SERF-MEG recording under pattern-reversal visual stimulation; (ii) Signal preprocessing, which includes baseline correction, band-pass filtering, bad-segment rejection, and independent component analysis (ICA) for artifact removal; and (iii) Feature and network analysis, which extracts functional connectivity measures and time-domain features (such as M100, M135, and peak-to-peak amplitude) for further graph-theoretical analysis.

This study was structured as a prospective case–control experiment to assess the feasibility and clinical usefulness of MEG in patients with optic nerve injury. The overall experimental workflow is illustrated in Figure 1. The study protocol received approval from the Ethics Committee of Qilu Hospital of Shandong University (Approval No. KYLL-202310-017-1) and was executed in alignment with the principles of the Declaration of Helsinki. Informed consent in writing was acquired from all individuals before enrollment.

### 2.2. Participants and Classification

This study enrolled both patients and healthy controls. The cohort comprised people diagnosed with ON, ION, or TON. All patients satisfied the recognized clinical diagnostic criteria for their specific diseases. Participants with associated central nervous system illnesses or serious retinal diseases were excluded. The healthy control group consisted of age- and sex-matched volunteers without a history of vision impairment, neurological illnesses, or ophthalmic diseases.

### 2.3. SERF-MEG Recording

MEG data were obtained utilizing a SERF-MEG system (SERF-MEG; LMEG-64A, Hangzhou Zero Magnetic Medical Equipment Co., Ltd., Hangzhou, China). The SERF-MEG system comprises a whole-head SERF sensor array, a small magnetic shielding system, a moveable examination bed, and a central control unit equipped with dedicated SERF-MEG acquisition software. Owing to its compact form, the system can be flexibly put in normal hospital surroundings and simply relocated or swiftly deployed when required.

The SERF sensors were uniformly dispersed on a rigid adult helmet. Each sensor operated in a dual-axis setup to detect both radial and tangential components of the brain-generated magnetic field. The system incorporates 64 physical SERF sensors for neural signal capture, all of which display a sensitivity better than 15 fT/Hz^1/2^ within the 1–30 Hz frequency range. In addition, three reference SERF sensors, positioned relative to the main detection array, were used to record external interference and assist common-mode noise reduction.

The small magnetic shielding system combines passive magnetic shielding and active magnetic field correction. Passive shielding was achieved using a three-layer permalloy cylinder shield, while the active compensating system consisted of specially designed cylindrical coils, low-noise current sources, and computer-controlled software. As a result, the background magnetic field within a radius of 50 cm from the center of the sensor array was maintained below ±0.5 nT.

Prior to the trial, individuals completed a standardized preparation procedure, including removal of all metallic items. After entering the shielded chamber, the participant’s head was stabilized within the helmet. Following system calibration, the experimental paradigm was launched and data gathering commenced.

A pattern-reversal visual stimulation paradigm was adopted. The stimulus consisted of an equal number of black and white squares presented on a display. The black and white squares quickly flipped polarity (black to white and white to black) without any change in overall luminance. The reverse rate was set at 2.0 reversals per second (r/s), equivalent to a temporal frequency of 1.0 Hz, with each entire cycle including two reversals. Monocular stimulation was delivered, and a total of 150 stimulus cycles were recorded for each participant.

### 2.4. MEG Signal Preprocessing

Raw SERF-MEG data were preprocessed using a consistent methodology. First, a band-pass filter was applied to remove noise beyond the frequency range of interest, including low-frequency drifts and high-frequency interference. Second, a 50 Hz notch filter was utilized to decrease power-line noise. Third, denoising was performed using SPM12 software by combining independent component analysis (ICA) with principal component analysis (PCA) to identify and remove non-neural components, therefore effectively reducing corrupted data segments while maintaining genuine neural signals. Fourth, ICA was further employed to estimate and reduce cardiac and ocular artifacts. Finally, after alignment with stimulus event markers, stimulus-locked epochs were retrieved from −100 to 400 ms relative to stimulus start. Baseline correction was made utilizing the −100 to 0 ms period to eliminate DC offsets.

### 2.5. Feature Extraction

#### 2.5.1. Time-Domain and Global Brain Features

Based on the preprocessed data, time-domain and global brain characteristics were subsequently retrieved.

**Figure 2:**
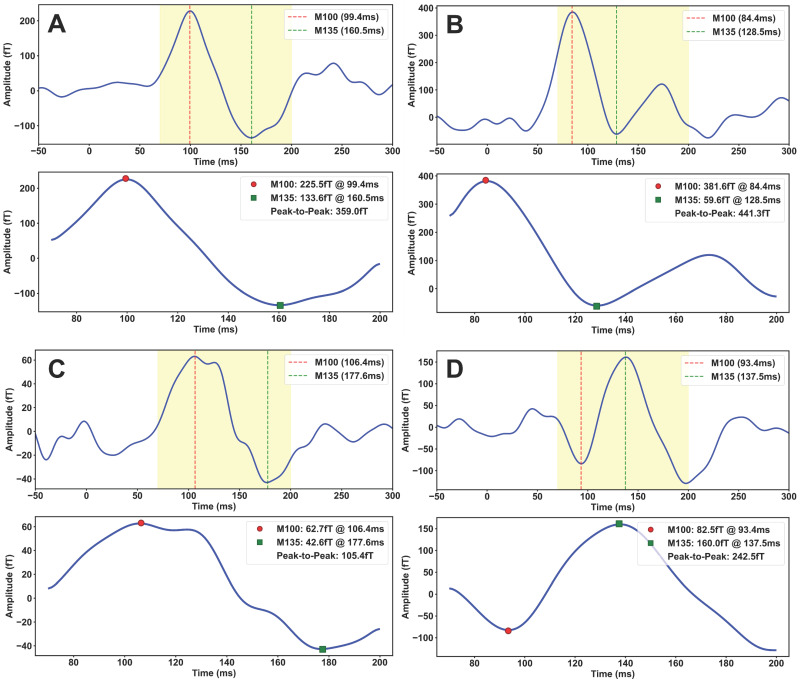
Representative waveforms and peak identification of visually evoked responses. Panels (**A**,**B**) show representative MEG-evoked waveforms from healthy controls, whereas panels (**C**,**D**) show representative waveforms from patients with optic nerve injury. The red dashed lines indicate the M100 peak latency, and the green dashed lines indicate the M135 peak latency. The yellow shaded area denotes the analysis time window (70–200 ms). The lower panels illustrate the corresponding peak detection results, with red and green dots marking the identified M100 and M135 peaks, respectively.

(1) Classical time-domain features:

These comprised peak amplitudes and latencies of the M100 and M135 components, peak-to-peak amplitude (Figure 2A,C), mean amplitude across all channels, and its variability. Within the response window of 70–200 ms, two main stimulus-evoked peaks with opposite polarities (M100 and M135) were automatically detected. For each component, peak amplitude and latency were extracted. To reduce statistical bias caused by peak jitter or peak misalignment across trials (Figure 2B,D), the peak-to-peak amplitude between M100 and M135 was additionally measured to evaluate the overall strength and temporal features of the visually elicited response. This metric indicates the degree of synchronous activation in the visual cortex in response to visual stimuli. Reduced amplitude or prolonged latency may indicate poor signal conduction throughout the visual pathway or delayed cortical processing.

(2) Global Field Power (GFP).

GFP was determined as the variance of the multichannel signals at each time point, reflecting the overall strength of the neural response. GFP was defined as:

$$\mathrm{GFP}\left(t\right)=\sqrt{\frac{1}{N}\sum_{i=1}^{N}{\left(x_{i}\left(t\right)-\bar{x}\left(t\right)\right)}^{2}}$$

where *N* denotes the number of channels, $\bar{x}\left(t\right)$ represents the instantaneous mean across all channels at time *t*, and $x_{i}\left(t\right)$ denotes the signal of the *i*-th channel at time *t*.

Because GFP does not depend on a reference electrode or the selection of a single channel, it is considered a robust metric for determining global cortical response intensity [30].

(3) Occipital sample entropy (Occipital_SampEn).

Occipital sample entropy was calculated based on channels located over the occipital cortex (O1, O2, and adjacent sensors). Sample entropy was defined as:

$$SampEn\left(m,r,N\right)=-ln\left(A/B\right)$$

where *m* denotes the embedding dimension (*m* = 2 in the present study), *r* denotes the similarity tolerance (*r* = 0.2 × SD), and SD represents the standard deviation of the signal. *A* and *B* denote the probabilities of matching template vectors of length *m* + 1 and *m*, respectively.

Higher SampEn values indicate increased signal complexity and reduced predictability of temporal patterns [31].

(4) Energy-Related Metrics.

To quantify the contribution of distinct temporal components to the total evoked response, signal energy within certain time periods was estimated as the time integral of the squared signal:

$$E=sum_{\left(t=t1\right)}^{\left(t2\right)}x{\left(t\right)}^{2}$$

The relative contribution of the M100 and M135 components to the total response energy was further calculated as:

$$Energy_{\left(ratio\right)}=E_{\left(component\right)}/E_{\left(total\right)}$$

where $E_{\left(component\right)}$ denotes the energy of a specific evoked component, $x\left(t\right)$ represents the stimulus-locked MEG time-series signal, and t1 and t2 denote the onset and offset of the corresponding component time window.

In the present investigation, the M100 time window was defined as 70–130 ms, while the M135 time window was established as 130–180 ms. Energy measures and their ratios represent the concentration of brain activity and changes in prominent response components, and have been employed in various MEG/electroencephalography (EEG) functional evaluation investigations [32].

(5) Methodological Comparison.

Two techniques were employed for feature extraction. First, the maximum peak-to-peak channel (MaxP2P) approach picked the channel having the biggest peak-to-peak amplitude within the response frame, representing features from the strongest responding channel. Second, the average across all channels (Avg) technique determined the mean value across all valid channels, demonstrating the overall consistency of cortical responses. Differences between these two methodologies were further studied in future methodological comparisons.

#### 2.5.2. Functional Connectivity Analysis

To investigate the impact of optic nerve injury on information transfer at the network level, multiple phase-based functional connectivity metrics were computed within the stimulus-related time window (0–200 ms), including coherence (COR), phase lag index (PLI), and weighted phase lag index (wPLI).

Among these measures, wPLI weights the imaginary component of phase discrepancies, thereby assessing the stability of phase lead–lag interactions between brain areas. Compared with conventional PLI, wPLI greatly lowers false synchrony produced by volume conduction and common noise sources, and displays increased statistical sensitivity [19]. Accordingly, wPLI was used as the key functional connectivity metric in this work to examine effective phase coupling changes in the visual cortex following optic nerve injury.

wPLIij=EImeiΔϕijtEImeiΔϕijt

In this context, $\Delta \phi_{ij}(t)\;$ denotes the instantaneous phase difference between nodes *i* and *j* at time *t*; *Im* (*·*) denotes the imaginary part; and *E*[*·*] represents averaging across time or trials.

Functional connectivity matrices were built based on 64 channels, and graph-theoretical methods were employed to generate numerous topological metrics to analyze network reorganization from an efficient “small-world” architecture toward less efficient or more randomized topologies [22].

(1) Global efficiency (GE), demonstrating the entire capacity for information integration across the network;

$$E_{global}=\frac{1}{N\left(N-1\right)}\sum_{i\neq j}\frac{1}{d_{ij}}$$

where $d_{ij}$ denotes the shortest path length between nodes *i* and *j*, and *N* denotes the number of network nodes.

(2) Local efficiency (LE), characterizing information transfer efficiency within local subnetworks;

$$E_{local}=\frac{1}{N}\sum_{i}E_{global}\left(G_{i}\right)$$

where $G_{i}\;$ represents the subgraph composed of the direct neighbors of node *i*.

(3) Mean connection strength (Strength_mean), evaluating the overall level of synchronization;

$$Strength_{mean}=\frac{1}{N}\sum_{i}\sum_{j}w_{ij}$$

where $w_{ij}$ denotes the connection weight between nodes *i* and *j*.

(4) Clustering coefficient (CC), reflecting the tendency of nodes to form local clusters;

$$C_{i}=\frac{1}{k_{i}\left(k_{i}-1\right)}\sum_{j,h}{\left(w_{ij}\;w_{ih}\;w_{jh}\right)}^{1/3}$$

where $C_{i}$ denotes the clustering coefficient of node *i*, $k_{i}$ denotes the degree of node *i*, and $w_{ij}$ denotes the connection weight between nodes *i* and *j*.

(5) Assortativity, describing the tendency for nodes with similar properties to preferentially connect, which may indicate compensatory network reorganization.

$$r=\frac{\sum_{ij}\left(w_{ij}-\frac{s_{i}s_{j}}{\sum_{ij}w_{ij}}\right)s_{i}s_{j}}{\sum_{ij}\left(s_{i}\delta_{ij}-\frac{s_{i}s_{j}}{\sum_{ij}w_{ij}}\right)s_{i}s_{j}}$$

Here, *r* denotes the assortativity coefficient; $w_{ij}$ denotes the connection weight between nodes *i* and *j*; $s_{i}=\sum_{i}w_{ij}$ denotes the weighted degree of node *i*; $s_{i}$ denotes the weighted degree of node *j*; $\delta_{ij}$ denotes the Kronecker delta (equal to 1 when *i* = *j*, otherwise 0); and $\sum_{ij}w_{ij}$ denotes the total sum of edge weights in the network.

These measurements were used to analyze moves from an efficient “small-world” architecture toward less efficient or more randomized network configurations.

To further analyze visual-pathway-specific connection changes, channels O1, O2, and Oz—corresponding to the occipital cortex and primary visual cortex—were selected as seed regions. Functional connectivity between these occipital seeds and all other channels was estimated, with an emphasis on three pathways: occipital–parietal, occipital–temporal, and intra-occipital connections.

### 2.6. Statistical Analysis

All statistical analyses were performed using Python 3.9, using the pandas, NumPy, and SciPy libraries. Absolute values of amplitude-related features (e.g., M100 and M135 amplitudes) were employed for descriptive statistics. Group differences were tested using analysis of variance (ANOVA) and *t*-tests, with effect sizes reported accordingly. Pearson correlation analysis was used to analyze correlations among extracted features and to compare the consistency between the MaxP2P and Avg feature extraction methodologies.

## 3. Results

### 3.1. Clinical Characteristics

A total of 43 patients and 43 healthy controls were included in the present investigation. The patient cohort includes 16 people with ON, 13 with ION, and 14 with TON. There were no significant differences between the patient and control groups in terms of age or sex distribution. All subjects successfully completed MEG recordings and subsequent feature extraction, and the overall data quality was deemed sufficient for future analysis.

### 3.2. Global Electrophysiological Differences

In terms of GFP, the patient group had considerably lower values than the healthy control group (36.9 ± 15.1 fT vs. 57.8 ± 17.9 fT; ANOVA: F = 34.22, *p* < 0.01), indicating a marked drop in the overall amplitude of cortical visual responses. In addition, Occipital_SampEn was substantially higher in patients compared with controls (0.60 ± 0.07 vs. 0.53 ± 0.05; F = 27.75, *p* < 0.01), suggesting increased instability and reduced regularity of neural response patterns in the visual cortex. Together, our findings indicate severe electrical anomalies at the cortical level in individuals with optic nerve injury, impacting not only individual response components but also the general features of visual cortical activity.

### 3.3. Time-Domain Feature Analysis

**Figure 3:**
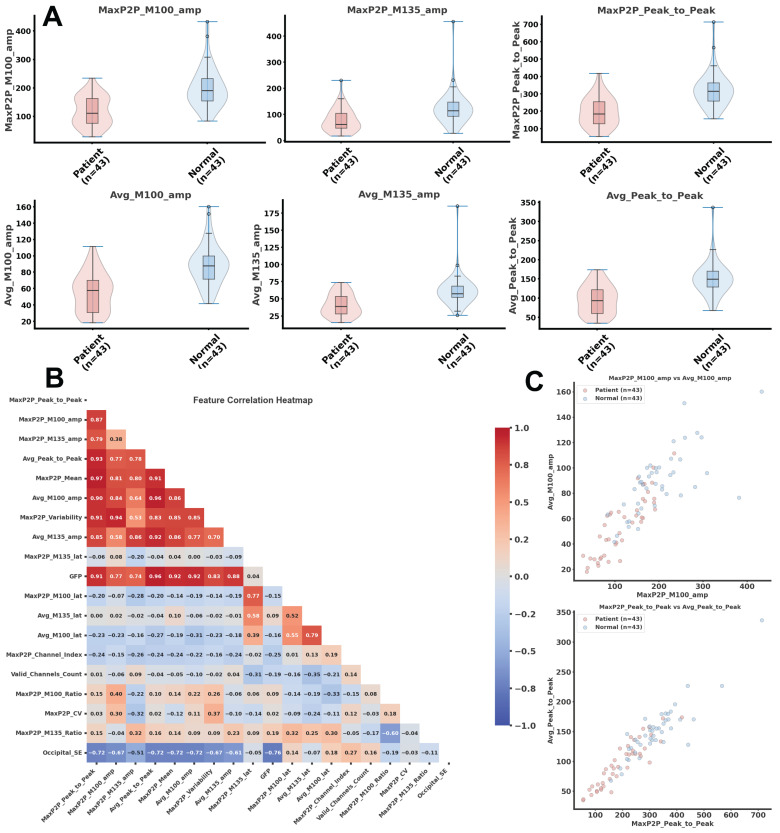
Time-domain features and methodological consistency analysis comparing patients with optic nerve injury and healthy controls. (**A**) Comparison of the amplitudes of important time-domain components (M100, M135, and peak-to-peak) between the two groups. Red signifies the sick group and blue shows the healthy control group. Results produced from two feature extraction approaches—MaxP2P and Avg—are shown. (**B**) Correlation heatmap of time-domain characteristics. Color intensity denotes the magnitude of the Pearson correlation coefficient, with red representing positive correlations and blue indicating negative correlations. (**C**) Scatter plots showing the sample-wise distribution and agreement between the two feature extraction methods.

As illustrated in Figure 3A, comparable abnormalities were seen in the major visual components M100 and M135, characterized by lower amplitudes and longer M100 latency in the patient group. For M100 amplitude, patients exhibited considerably lower values than controls using both feature extraction methodologies (MaxP2P: 117.5 ± 52.3 fT vs. 200.1 ± 71.2 fT, *p* < 0.01; Avg: 55.2 ± 23.8 fT vs. 88.8 ± 25.5 fT, *p* < 0.01). M100 latency was considerably prolonged in patients (MaxP2P: 104.2 ± 19.8 ms vs. 96.2 ± 13.6 ms, *p* < 0.01; Avg: 108.3 ± 7.5 ms vs. 102.6 ± 7.4 ms, *p* < 0.01). Similarly, M135 amplitude was considerably reduced in the patient group (MaxP2P: 77.8 ± 45.9 fT vs. 124.4 ± 66.2 fT, *p* < 0.01; Avg: 41.0 ± 16.4 fT vs. 62.3 ± 23.7 fT, *p* < 0.01), although no significant group difference was detected for M135 latency. These results indicate that both M100 and M135 amplitudes function as sensitive disease-related indicators, but latency variations are predominantly confined to the M100 component.

Regarding the overall peak-to-peak amplitude, patients revealed substantially lower values than healthy controls (MaxP2P: 195.4 ± 85.0 fT vs. 324.5 ± 100.9 fT, *p* < 0.01; Avg: 96.2 ± 38.4 fT vs. 151.1 ± 44.3 fT, *p* < 0.01). In addition, both the mean amplitude and amplitude variability were considerably reduced in patients (*p* < 0.01), showing deficits in both response strength and dynamic modulation of cortical activity. Energy-related measures further characterized the response pattern. The M100 energy ratio (M100_Ratio) was substantially lower in patients than in controls (0.30 ± 0.16 vs. 0.39 ± 0.16, *p* < 0.01), although no significant difference was seen for the M135 energy ratio. These data show that attenuation of the early M100 component reflects a major electrophysiological characteristic associated with optic nerve injury.

### 3.4. Feature Correlation Analysis

The findings of correlation analyses are displayed in Figure 3B. Strong consistency was detected among amplitude-related metrics: GFP showed very significant correlations with numerous amplitude parameters, including M100 amplitude, M135 amplitude, and peak-to-peak amplitude (all r > 0.9). Latency-related measurements indicated moderate correlations (M100 latency vs. M135 latency: r = 0.79). A significant degree of agreement was also established between the two feature extraction algorithms (Figure 3C). Correlation coefficients for amplitude measures above 0.8 (M100 amplitude: r = 0.84; M135 amplitude: r = 0.86), with the best correlation reported for peak-to-peak amplitude (r = 0.93). In contrast, latency measures showed only minor associations (M100 latency: r = 0.55; M135 latency: r = 0.58). These data indicate that both feature extraction methodologies reliably capture group-level differences, although amplitude-based metrics demonstrate superior stability and consistency. Consequently, amplitude-related metrics may provide more robust and clinically useful biomarkers for detecting visual cortex dysfunction.

### 3.5. Overall Alterations in Network Topology

At the global functional connectivity level, the patient group displayed a considerable loss in network integration efficiency coupled by weaker local clustering features. Analyses based on COR and phase-lag measures (PLI/wPLI) consistently demonstrated significantly decreased GE in patients compared with healthy controls (COR: *p* < 0.01, d = −0.50; PLI: *p* < 0.01, d = −0.81; wPLI: *p* < 0.01, d = −1.33), indicating impaired large-scale information transfer across the brain network. Concurrently, a significant fall in the weighted CC was detected (wPLI: *p* < 0.01, d = −0.54), demonstrating weaker functional connectivity within local network modules. Together, these data reflect a transition of the cortical network from an efficient small-world organization toward a less efficient and more fragmented topology in individuals with optic nerve injury.

Given the various functional functions of brain oscillatory frequency bands in visual information processing, functional connection networks were thoroughly studied across multiple frequency ranges. Previous studies have shown that alpha-band activity (8–13 Hz) is closely associated with cortical inhibition, feedback modulation, and interregional integration within the visual system, whereas beta-band activity (13–30 Hz) is more strongly involved in higher-order visual integration, attentional control, and inter-areal coordination. Moreover, impairments in alpha- and beta-band functional connectivity have been found as sensitive indicators of cortical network rearrangement in demyelinating illnesses such as MS and ON. Based on an initial multi-band comparison, the present study primarily focuses on network topology modifications in the alpha and beta bands, seeking to define frequency-specific network reconfiguration associated with optic nerve injury (Table 1).

(1) Alpha-Band Network Alterations

In the alpha band (8–13 Hz), the most substantial functional connectivity abnormalities were identified in the patient group. Networks created using the wPLI exhibited considerable reductions in both Strength_mean and GE, with drops of around 24% and 27%, respectively, relative to healthy controls. These differences were extremely statistically significant (Strength_mean: *p* < 0.01, d = −1.14; GE: *p* < 0.01, d = −1.33).

**Figure 4:**
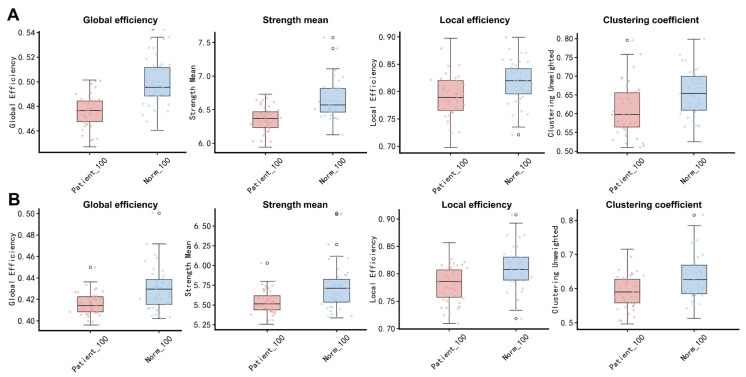
Functional connectivity alterations in patients with optic nerve injury in the alpha and beta frequency bands. (**A**) Group comparisons of global efficiency, local efficiency, mean connection strength, clustering coefficient, and assortativity in the alpha band. (**B**) Group comparisons of the same network metrics in the beta band. Red indicates the patient group, and blue indicates the healthy control group. The central line in each box represents the median, and the box boundaries indicate the interquartile range.

In addition, both LE and the CC were considerably reduced (*p* < 0.01, d = −0.65; *p* < 0.01, d = −0.66), indicating disruption of local information transfer and diminished node clustering. In contrast, assortativity was considerably increased (*p* < 0.01, d = 0.81), suggesting compensatory topological remodeling defined by a more uniform pattern of interregional connectivity (Figure 4A).

(2) Beta-Band Network Alterations

In the beta band (13–30 Hz), patients likewise demonstrated broad network impairment. wPLI-based analyses showed a significant loss in GE (*p* < 0.01, d = −0.86), accompanied by lower LE and CC (*p* < 0.01, d = −0.74; *p* < 0.01, d = −0.73). At the same time, Strength_mean was significantly reduced (*p* < 0.01, d = −0.81), indicating weaker interregional synchronization and overall network connectivity in the beta band. Similar to the alpha band, assortativity was significantly increased (*p* = 0.02, d = 0.51), again pointing to compensatory topological reconfiguration within the functional network (Figure 4B).

### 3.6. Visual-Pathway-Specific Connectivity Alterations

**Figure 5:**
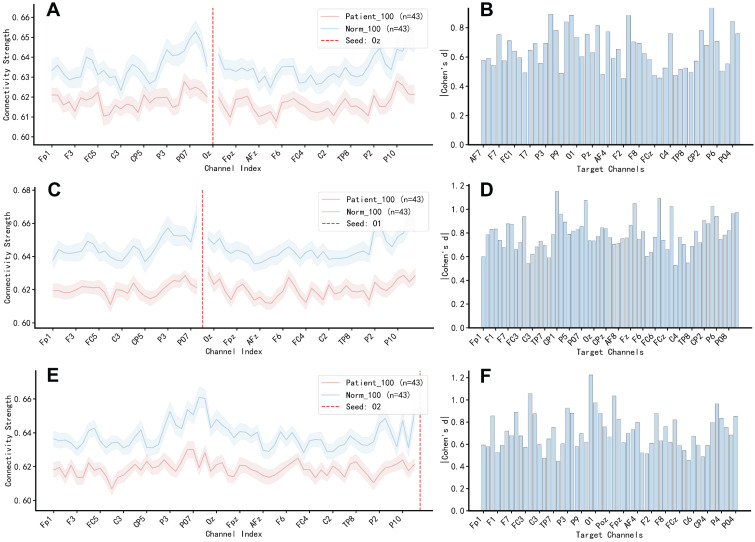
wPLI-based occipital-seed-based functional connectivity analysis. (**A**,**C**,**E**) Line plots showing the spatial distribution of functional connectivity for the Oz, O1, and O2 seed channels, respectively. The blue line represents the healthy control group, and the red line represents the patient group. The red dashed line indicates the location of the seed channel. (**B**,**D**,**F**) Histograms of channels showing significant between-group differences for the Oz, O1, and O2 seeds, respectively.

Seed-based analyses using wPLI were performed with occipital channels O1, O2, and Oz as seeds to assess visual-pathway-specific phase coupling differences between patients and healthy controls. As demonstrated in Figure 5, patients exhibited severe decreases in connection within occipital-centered visual networks, particularly affecting occipito-parietal and occipito-temporal pathways. The greatest substantial effects were detected in connections projecting from O1/O2 into parietal and temporal areas (Table 2 and Table S1).

(1) Reduced Occipito-Parietal Connectivity

Significant decreases were detected in many linkages linking occipital and parietal regions. Notably, connections such as O1–CP1 (*p* < 0.01, d = −1.15), O1–P4 (*p* < 0.01, d = −1.02), O2–P2 (*p* < 0.01, d = −0.97), and Oz–CPz (*p* < 0.01, d = −0.82) showed highly significant declines. These findings reveal markedly reduced phase synchronization between early visual cortex (V1/V2) and parietal integration areas, reflecting impaired efficiency of visual information transfer toward spatial processing regions.

(2) Reduced Occipito-Temporal Connectivity

Several connections between occipital and temporal regions showed significant reductions, including O2–FT7 (*p* < 0.01, d = −0.89), O1–FT7 (*p* < 0.01, d = −0.87), O2–TP7 (*p* < 0.01, d = −0.75), and O1–TP7 (*p* < 0.01, d = −0.69). These findings indicate decreased phase synchronization between occipital visual areas and temporal cortical regions, suggesting altered functional interactions within visual-related networks.

(3) Reduced Intra-Occipital Coupling

Within the occipital cortex itself, patients demonstrated significantly lower wPLI values between occipital nodes (e.g., O1–Pz, O2–Pz, Oz–PO3; all *p* < 0.01, d ≤ −0.80), showing disturbed phase synchronization even within the visual cortical network. This data implies that functional coupling impairments extend beyond long-range pathways to impact local visual cortical circuitry.

## 4. Discussion

Using SERF-MEG, the present study investigated neural network reconfiguration in patients with optic nerve injury at both the level of time-domain evoked responses and frequency-specific functional connectivity. Our results demonstrated significant abnormalities in M100/M135 components, energy-related metrics, and alpha- and beta-band functional connectivity, indicating that optic nerve injury not only impairs cortical transmission and integration of visual signals but also induces frequency-specific network reorganization. These findings verify the sensitivity of MEG in detecting functional problems associated with optic nerve injury and underline its potential clinical utility as a novel technique for integrated structure–function assessment.

### 4.1. Optic Nerve Injury Is Associated with Altered Cortical Visual Responses

In the temporal domain, patients demonstrated substantial attenuation of early visual cortex responses associated with delayed signal transmission. Specifically, the M100 peak amplitude was significantly reduced in patients compared with healthy controls (MaxP2P: 117.5 ± 52.3 fT vs. 200.1 ± 71.2 fT, corresponding to an approximate 41% decrease), while M100 latency was significantly prolonged (104.2 ± 19.8 ms vs. 96.2 ± 13.6 ms, an average delay of approximately 8 ms). The overall peak-to-peak amplitude was also considerably reduced (by around 40%), indicating a major drop in the overall strength of cortical responses to visual stimulation. The extent of these changes is congruent with recent findings from VEP studies in ON and associated optic nerve diseases, which have indicated P100 amplitude reductions and latency delays [33,34]. Together, these findings indicate that optic nerve injury is associated with reduced cortical response magnitude and altered temporal dynamics in early visual processing.

In addition, the M100_Ratio was significantly lower in patients than in controls (0.30 ± 0.16 vs. 0.39 ± 0.16, corresponding to a reduction of approximately 23%), indicating that the contribution of early visual components to the overall cortical response was diminished within the stimulus-locked time window. This observation continues after peak amplitude reductions and provides evidence for a redistribution of cortical response energy, suggesting ongoing suppression of cortical integration efficiency rather than a temporary attenuation of signal amplitude alone.

Compared with conventional VEP, which generally relies on amplitude and latency data from single or limited channels, MEG allows multidimensional characterization of cortical responses through multichannel spatiotemporal analysis, including amplitude, latency, and energy distribution. In the present experiment, comparison between the MaxP2P and Avg approaches indicated that response attenuation was detectable not only in the most sensitive channels but also at the level of global cortical consistency. This pattern implies a system-wide reduction in visual cortex responsiveness rather than a specific defect. These findings are consistent with prior EEG and fMRI investigations indicating reduced cortical activity magnitude and spatial extent following visual pathway damage [35,36,37].

### 4.2. Optic Nerve Injury Is Associated with Frequency-Specific Functional Network Reorganization

Alpha- and beta-band oscillations play complementary roles in visual information processing and large-scale cortical communication [38,39]. Based on this functional framework, the present study focused on alpha- and beta-band functional connectivity and network structure.

Patients demonstrated notable reductions in global and local efficiency within the alpha band, alongside diminished mean strength and clustering coefficient, exhibiting medium-to-large effect sizes. These results align with diminished network integration and modified local topological structure within the visual network. The noted rise in assortativity indicates a transition towards favored connections among nodes with analogous degrees, although the functional significance of this alteration is yet to be established. In the beta band, patients exhibited diminished network efficiency alongside modified topological characteristics, indicating extensive network modifications within the visual system. Prior EEG and MEG investigations have documented anomalies in alpha- and beta-band networks associated with visual pathway diseases, including optic neuritis and multiple sclerosis [40,41]. The current findings augment these observations by revealing frequency-specific network disparities under stimulus-evoked settings, suggesting that optic nerve injury correlates with band-specific alterations in functional network organization, including local cortical response anomalies.

Notably, seed-based analyses indicated dramatically reduced phase synchronization across occipital-centered visual networks, particularly along occipito-parietal and occipito-temporal connections, with O1, O2, and Oz functioning as main seed nodes. These connection patterns are anatomically compatible with posterior visual pathways linking occipital areas with parietal and temporal cortices. The loss in phase coupling reflects altered interregional coordination within stimulus-evoked visual networks. Such convergent multiscale changes enhance the sensitivity of MEG for detecting cortical-level functional impairments associated with optic nerve damage.

### 4.3. Clinical Relevance, Limitations, and Future Perspectives of SERF-MEG

The SERF-MEG system has several practical advantages, including the absence of cryogenic cooling needs, compact system architecture, and high sensitivity, facilitating steady operation under normal clinical circumstances [11,42]. By integrating time-domain features, energy distribution metrics, and frequency-specific network properties, MEG allows a graded assessment of visual system function, spanning localized cortical responses to system-level network organization. Although the present study utilized a cross-sectional technique and consequently cannot directly show diagnostic or prognostic utility, the current findings suggest that MEG-derived metrics may provide complementary information regarding cortical functional alterations following optic nerve injury. Future investigations integrating MEG with OCT and structural or functional MRI will be necessary to further evaluate the sensitivity and specificity of MEG-derived metrics and to progress the establishment of an integrated structure–function framework for the visual system.

Several limitations of the present investigation should be addressed. First, the sample size was quite moderate, and no stratified analyses were performed among ON, ION, and TON subtypes. Larger cohorts will be necessary to validate disease-specific MEG signals. Second, the cross-sectional methodology prohibits investigation of dynamic correlations between MEG measurements, disease progression, and treatment response, underscoring the need for longitudinal follow-up studies. Third, only a checkerboard reversal paradigm was used, limiting assessment of higher-order visual processing. Moreover, data analysis was constrained to the sensor level, without source reconstruction, restricting precise anatomical localization and inference regarding interregional causal relationships. These limitations restrict comprehensive investigation of higher-order visual networks and complex cortical integration mechanisms. Future investigations including multiple stimulation protocols, source-level analysis, and multimodal imaging data (e.g., OCT and fMRI) will be critical for expanding understanding of structure–function links in optic nerve injury.

## 5. Conclusions

This study demonstrates that SERF-based MEG is capable of capturing cortical functional alterations and frequency-specific network changes following optic nerve injury. Owing to its high spatiotemporal resolution and multiband analytical capability, MEG provides a multidimensional characterization of visual system dysfunction beyond conventional structural imaging and single-channel electrophysiological assessments. Although the present findings are based on a cross-sectional design, they suggest that MEG-derived time-domain and network-level features may have translational relevance for the functional evaluation of visual pathway disorders. Future studies integrating MEG with multimodal imaging and longitudinal follow-up will be necessary to further clarify its clinical applicability and to define its potential role in disease monitoring and biomarker development in neuro-ophthalmic conditions.

## Figures and Tables

**Table 1:** Group differences in network topology metrics between patients and healthy controls in the alpha and beta frequency bands.

Frequency Band	Metric	Control Group	Patient Group	*p*-Value	Cohen’s d
Alpha band	Global efficiency	0.50 ± 0.02	0.48 ± 0.01	<0.01	–1.33
	Strength_mean	6.64 ± 0.30	6.36 ± 0.19	<0.01	–1.14
	Local efficiency	0.82 ± 0.04	0.79 ± 0.04	<0.01	–0.65
	Clustering coefficient	0.65 ± 0.06	0.61 ± 0.07	<0.01	–0.66
	Assortativity	–0.27 ± 0.08	–0.20 ± 0.09	<0.01	+0.81
Beta band	Global efficiency	0.43 ± 0.02	0.42 ± 0.01	<0.01	–0.86
	Strength_mean	5.73 ± 0.30	5.54 ± 0.15	<0.01	–0.81
	Local efficiency	0.81 ± 0.04	0.78 ± 0.03	<0.01	–0.74
	Clustering coefficient	0.64 ± 0.07	0.59 ± 0.05	<0.01	–0.59
	Assortativity	–0.24 ± 0.11	–0.19 ± 0.08	<0.01	–0.73

**Table 2:** Occipital-seed-based wPLI connectivity differences between patients and healthy controls.

Seed	Affected Network	Representative Target Channels	Cohen’s d	No. of Significant Connections
Oz	Occipito-parietal	P3, P4, PO3, CPz	−0.63 ~ −0.94	14
	Occipito-temporal	T7, TP7	−0.65 ~ −0.75	4
	Occipito-frontal	F7, F4, FC3	−0.59 ~ −0.89	12
	Inter-occipital	O1, O2	−0.73 ~ −0.76	2
O1	Occipito-parietal	CP1, P3, P4, PO3, PO7	−0.62 ~ −1.15	28
	Occipito-temporal	T7, TP7, FT7	−0.69 ~ −0.88	6
	Occipito-frontal	Fz, F3, F7, FC1, FC4	−0.60 ~ −1.09	22
	Inter-occipital	O2, Oz	−0.73 ~ −0.97	2
O2	Occipito-parietal	Pz, P3, P4, PO3	−0.60 ~ −1.22	18
	Occipito-temporal	T7, TP7	−0.65 ~ −0.75	4
	Occipito-frontal	FC1, F4, AF3	−0.57 ~ −1.06	16
	Inter-occipital	O1, Oz	−0.76 ~ −0.97	2

## Data Availability

The datasets generated and analysed during the current study are not publicly available due to patient privacy concerns but are available from the corresponding author on reasonable request.

## Supplementary Materials

The following supporting information can be downloaded at: https://www.mdpi.com/article/10.3390/bioengineering13060694/s1, Table S1: Seed-based connectivity differences.

## Abbreviations

The following abbreviations are used in this manuscript:

MEG	Magnetoencephalography
SERF-MEG	Spin-exchange relaxation-free magnetoencephalography
VEP	Visual evoked potential
OCT	Optical coherence tomography
fMRI	Functional magnetic resonance imaging
EEG	Electroencephalography
ON	Optic neuritis
ION	Ischemic optic neuropathy
TON	Traumatic optic neuropathy
NMOSD	Neuromyelitis optica spectrum disorder
MS	Multiple sclerosis
V1	Primary visual cortex
V2	Secondary visual cortex
GFP	Global field power
Occipital_SampEn	Occipital sample entropy
wPLI	Weighted phase lag index
MaxP2P	Maximum peak-to-peak amplitude
Avg	Whole-channel average
GE	Global efficiency
LE	Local efficiency
CC	Clustering coefficient
Strength_mean	Mean connection strength
